# Diagnosis, quality of life, and treatment of patients with Hunter syndrome in the French healthcare system: a retrospective observational study

**DOI:** 10.1186/s13023-015-0259-0

**Published:** 2015-04-12

**Authors:** Nathalie Guffon, Bénédicte Heron, Brigitte Chabrol, François Feillet, Vincent Montauban, Vassili Valayannopoulos

**Affiliations:** Centre de Référence des Maladies Héréditaires du Métabolisme, Hospices Civils de Lyon Hôpital Femme Mère Enfants, Bron, France; Service de Neurologie Pédiatrique, Centre de Référence des Maladies Lysosomales, CHU Trousseau, APHP, Paris, France; Service de Neurologie Pédiatrique, Centre de Référence des Maladies Héréditaires du Métabolisme. Hôpital d’Enfants, CHU La Timone, Marseille, France; Centre de Référence des Maladies Héréditaires du Métabolisme, Service de Médecine Infantile, CHU Brabois Enfants, Vandoeuvre les Nancy, France; Shire, 88 rue du Dôme, Boulogne Billancourt, 92100 France; Centre de Référence Maladies Métaboliques de l’enfant et de l’adulte, Hôpital Universitaire Necker-Enfants Malades et Institut IMAGINE, Paris, France; Current address: Baxter Healthcare, 6 Avenue Louis Pasteur - BP 56, Maurepas, 78311 France

**Keywords:** Hunter syndrome, Lysosomal storage disease, Iduronate-2-sulfatase, Enzyme replacement therapy, Quality of life

## Abstract

**Background:**

Mucopolysaccharidosis II (MPS II) is associated with a broad spectrum of chronic and progressive, life-limiting symptoms. Idursulfase is approved for MPS II enzyme replacement therapy (ERT) in over 50 countries. This retrospective study evaluated the MPS II burden, organization of clinical care, and effects of idursulfase treatment on the disease in France.

**Methods:**

MPS II patients who had received idursulfase ERT in the French healthcare system were enrolled. In addition to clinician and patient questionnaires, the Clinical Global Impression-Improvement (CGI-I); Patient Global Impression-Improvement (PGI-I); KIDSCREEN-27, and EuroQoL-5D for adult patients scales were used to assess quality of life (QoL) and efficacy.

**Results:**

Fifty-two patients were enrolled from 5 sites in France. The majority of patients (69.2%) presented a severe MPS II phenotype with progressive neurocognitive impairment. Major impacts on QoL were apparent, with at least 1 member of the family having to reorganize working hours (45.5%) or to stop working (22.7%). KIDSCREEN-27 and EuroQoL-5D scale scores were well below those for referent (control) populations. Most families (70.0%) experienced a diagnostic delay of at least 3 years after the initial observation of symptoms. The MPS II diagnosis was often delivered without adequate sensitivity, psychological support, or comprehensive information about the disease. The study population had received a mean of 3.8 ± 1.3 years ERT. Forty-four percent of patients with the attenuated phenotype (without progressive neurocognitive impairment) showed symptom improvement during both the first year (Period 1) and from the end of the first year of treatment to “the present” (Period 2), as measured by CGI-I/PGI-I. 30.3% and 9.1% of severe patients experienced symptom improvement during Periods 1 and 2, respectively, while 63.6% and 51.5% displayed no change. The most common adverse reactions reported were skin rash and other infusion-associated reactions.

**Conclusions:**

MPS II adversely affects multiple domains of QoL for patients and families, requiring multiple healthcare services and social aid programs. The majority of patients with either phenotype experienced either improvement or stability in their symptoms during the first year of ERT, but this was clearly less so for patients with the severe phenotype after the first year of treatment.

## Background

Mucopolysaccharidosis II (MPS II), also called Hunter syndrome, is a rare X-linked recessive lysosomal storage disease caused by iduronate-2-sulfatase enzyme deficiency, leading to accumulation of the glycosaminoglycans, heparan and dermatan sulfate. The abnormal deposition of glycosaminoglycans results in dysfunction of multiple organs and systems and causes a broad spectrum of chronic and progressive, life-threatening symptoms [1]. The presentation and progression of MPS II is variable. Several studies have noted significant differences between attenuated and more severe phenotypes in terms of age at diagnosis, age at death, and disease manifestations. The severe phenotype is more common, affecting about two-thirds of patients with MPS II, and is characterized by progressive cognitive impairment and developmental regression, with death usually occurring in the second decade of life. Patients with the attenuated phenotype have no severe cognitive impairment [2], but commonly suffer from joint stiffness and contractures, cardiac disease and respiratory infection, and may rarely display non-progressive cognitive impairment.

Children with either phenotype generally appear normal at birth. Young et al. noted that the average age of onset of symptoms was 2.5 years of age for severe patients and 4.3 years of age for attenuated patients; the average age of death was 11.8 years and 21.7 years, respectively [3]. Some patients with the attenuated form may not experience symptoms until late childhood or early adolescence and may have near normal life expectancy, while others may experience significant morbidity and disability, including mild to moderate learning difficulties [2]. Indeed, the number and somatic manifestations can vary widely among patients within either phenotypic group, and the 2 phenotypes should be considered a continuum rather than 2 distinct groups. The diverse symptoms and functional deficits associated with MPS II may severely impair the quality of life (QoL) of patients and their caregivers or families [4]. However, the total burden of MPS II, including healthcare, social, and economic domains, is not well studied.

Recombinant human idursulfase (Elaprase®, Shire, Lexington, MA) is approved in over 50 countries for treatment of patients with MPS II. Regulatory agencies approved idursulfase as enzyme replacement therapy (ERT), based on the results of a pivotal phase 2 and 3 randomised, double-blind, placebo-controlled clinical trial in 96 patients with MPS II, all with the attenuated phenotype, aged 5 to 31 years [5]. An expert panel has stated that all previously diagnosed patients for whom there is an expectation that ERT will alter the course of somatic involvement should be candidates for treatment, even if cognitive impairment is already evident [6]. The expert panel also emphasized the necessity of timely, individualized treatment. Generally, ERT should be offered to all patients older than 5 years with the attenuated phenotype; however, recent studies in children younger than 5 years showed that results were similar to those from the pivotal trial [7,8]. The importance of early intervention with ERT is supported by data from recent studies [9]. A study from Poland showed that early intervention of ERT may markedly slow or prevent some irreversible manifestations of MPS II, including coarse facial features, joint disease, and cardiac function [10]. For patients with severe cognitive decline and physical impairment, it is recommended that the decision to initiate ERT (or not) be made by the treating physicians, the institution’s ethics committee, and the patient’s family [11]. In addition, the decision of whether to initiate ERT must include consideration of the total burden of MPS II and its treatment on QoL, including economic, occupational, and lifestyle factors, as well as clinical measures.

## Objectives

The aims of this study were to evaluate the burden of MPS II on the QoL of patients and families and on the healthcare and social services system in France, to document the organization and quality of clinical management of MPS II, and to describe the effects of idursulfase ERT on disease progression as perceived by clinicians and patients.

## Methods

A cross-sectional study design was used to collect patient and physician information using questionnaires and scales for a national sample of patients with MPS II treated with idursulfase in France. Two periods were evaluated: Period 1 started at baseline of initial idursulfase treatment and ended after 1 year of treatment; Period 2 began at the end of the first year of idursulfase treatment and extended to the present (time of study evaluation), and was required to be >2 years. The duration of the data collection period for this study was 12 months from February 1, 2011 to 31 January 2012. Subjects were requested to provide retrospective data when necessary.

### Patient population

Patients who had a confirmed diagnosis of MPS II, were receiving or had received idursulfase, and were being treated by the French healthcare system only were enrolled. Eligible patients were identified by the specialized French Reference Centers for Inherited Metabolic Disease and Lysosomal Diseases. Those judged eligible were mailed invitations to participate and were sent follow up reminders if no response was obtained after the first mailing. Patients/caregivers who accepted the invitation were required to provide their informed, written consent for their medical data to be used anonymously (written consent for patients aged 18 years and older, written consent of at least one of the parents for patients younger than 18 years or adult patients with severe cognitive impairment, or by his/her legal representative) for inclusion in the study in conformance with the provisions of the *Commission Nationale de L’Informatique et des Libertés*. Because this study was observational in design, and did not involve medical intervention (beyond the usual treatment received by the patients) or physical or psychological examination, it was not subject to the provisions of the *Comité de Protection des Personnes*. The investigators did not receive compensation for their participation in the study.

### Definitions of phenotypes

For the purposes of this study, patients were categorized by the 2 phenotypes of MPS II, severe (with progressive neurocognitive impairment) and attenuated (without progressive cognitive impairment), defined as follows:

- Severe phenotype—with neurocognitive regression (most frequent). The first symptoms usually occur during the first year of life and gradually increases before the age of 2–3 years, with symptoms including coarse facial features, enlarged liver and spleen, stiff joints and contractures, dysostosis multiplex, upper airway obstruction, hearing loss, cardiac valve disease, and profound, progressive neurological involvement. Death usually occurs in the second decade of life.
- Attenuated phenotype—mild mental retardation may be present, but without cognitive decline. The attenuated forms of MPS II manifest in a wide variety of ways. The symptoms are usually less marked than the severe phenotype, and they usually appear later in childhood or adolescence. Despite the absence of cognitive impairment; somatic symptoms such as hearing loss, glaucoma, retinopathy, dysostosis multiplex, carpal tunnel syndrome, spine compression, valvulo- and cardiomyopathy, upper respiratory tract dysfunction, respiratory insufficiency, hepatosplenomegaly, chronic diarrhoea, and short stature, are common. Patients may live until adulthood [2,12].

### Study questionnaires and scales

Clinician and patient questionnaires were administered at baseline, following enrollment of eligible patients, to gather demographic and disease data on patients, as well as information on the experience of patients and their families of their treatment, and how they cope with the burdens of the disease. Note that the questionnaires asked respondents to recollect current and historical data. The clinician questionnaires included: MPS II history, including date of diagnosis, specialty and setting of physician who made the diagnosis, date of first symptoms, family history of the disease; clinical characteristics of MPS II, including body systems affected and severe events documented; criteria used to determine patient eligibility for treatment; treatment history, including onset, dose and frequency of idursulfase treatment and method of administration (peripheral intravenous, or central vein catheter); frequency of medical consultations; and the roles of the reference center and secondary center for follow up procedures. Items addressed in the patient questionnaire included the circumstances of the first observation of MPS II signs and symptoms; the time from the observation of first signs to formal diagnosis; circumstances of the MPS II diagnosis; names and locations of the reference and secondary treatment centers; the distances between the patient’s home and the reference and secondary centers; modes of transportation used by the patient to reach the center where idursulfase was administered; level of transport coverage; and affiliation to patient associations and their role.

In addition to the clinician and patient questionnaires, the following 4 instruments for measurement of QoL and the effects of idursulfase treatment were used:

- **The Clinical Global Impression-Improvement (CGI-I) and Patient Global Impression-Improvement (PGI-I)** are scales that require the clinician (CGI-I) or the patient/caregiver (PGI-I) to assess whether and how much the patient’s illness has improved or worsened relative to a baseline state at the beginning of the treatment or time period with a choice of 7 responses: very much improved, much improved, minimally improved, no change, minimally worse, much worse, or very much worse [13]. The CGI-I and PGI-I were measured for 2 periods. Period 1 compared patient status at baseline or before the initiation of idursulfase treatment with that 1 year later. Period 2 compared the patient’s status at 1 year after the start of idursulfase ERT with the present; Period 2 was required to be greater than 2 years in duration. For all CGI-I and PGI-I evaluations, respondents were asked to give ratings irrespective of whether they thought any change was due to the drug treatment.
- **KIDSCREEN-27:** This instrument is designed to assess children’s and adolescent’s subjective health and well-being in 5 dimensions: physical well-being (5 items), psychological well-being (7 items), autonomy and relationship with parents (7 items), social support (4 items), and school environment (4 items). These instruments were developed as self-reporting measures applicable for healthy and chronically ill children and adolescents, ages 8 to 18 years [14-17].
- **EuroQoL-5D-3L (EQ-5D):** This is a standardised instrument for use as a measure of health outcomes and comprises 5 dimensions: mobility, self-care, usual activities, pain/discomfort, and anxiety/depression, each with 3 response levels: no problems, some problems, and unable to/extreme problems [18]. The patients were also asked to rate their response for each dimension on a vertical visual analogue scale (VAS) of 0 (Worst imaginable health state) to 100 (Best imaginable health state).

The instruments used in the study were targeted for specific age groups. For patients less than 8 years of age, the PGI-I family and KIDSCREEN-27 were used; for those aged 8 to 18 years and considered incapable, the PGI-I family and the KIDSCREEN-27 family versions were used. For those 8 to 18 years who were considered capable, the PGI-I family version and self-rated KIDSCREEN-27 were used. For patients over 18 years, the PGI-I self-rated questionnaire and EQ-5D were used. Scores of KIDSCREEN-27 and EQ-5D were compared with those of reference (control) respondents in a general population of the same age and sex.

### Statistical methods

Descriptive statistics were used for qualitative and ordinal variables in terms of total numbers and frequency of each condition; quantitative variables were described in terms of number of responses, mean, standard deviation, minimum, maximum, and median. For comparative analysis of qualitative variables, the Pearson chi-square test was applied except when the theoretical total numbers were less than 5, in which case, the Yates’ continuity correction or Fisher’s exact test was used. For quantitative variables, Student *t* tests or variance analysis were used.

## Results

### Patient population

A total of 62 eligible patients with MPS II who had received, or were currently receiving, idursulfase treatment were identified at 6 French reference centers. Of this total, 10 eligible patients were not enrolled. Four patients were not approached because of the state of their health, and 2 were not approached due to their social circumstances. Four patients who were approached did not consent, although the reasons for this are not known. The remaining 52 patients at 5 reference centers who accepted the invitation were enrolled in the study. The patients were all males and the great majority (84.4%) of patients were minors, and 48 (92.3%) were living with their parents.

Completed questionnaires were obtained from 51 (98.1%) patients for the clinical section and family interview, 33 (63.5%) answered the QoL questionnaires, including 26 (50.0%) who responded to the KIDSCREEN-27 scale (5 child, and 21 parent versions), and 7 (13.5%) who completed the EQ-5D scale (5 for the self-rated versions, and 2 parent versions).

### Demographic and clinical characteristics of study population

Characteristics of the study population are presented in Table 1. The mean (SD) age was 12.4 ± 9.2 years (range 1 year to 51 years), and most patients were aged between 5 and 15 years. A family history of MPS II was present in 17 (32.7%) patients, while 31 (59.6%) respondents reported no family history of MPS II, and 4 (7.7%) said that they did not know. Prenatal diagnosis had been performed for only 1 patient.

**Table 1:** **Patient demographic and clinical characteristics**

**Characteristic**	**All patients (N = 52)**
Gender	
Male	52
Age, y^a^	
Mean (SD)	12.4 (9.2)
Median (min, max)	11.0 (1.3, 51.0)
Age, y, category, n (%)^a^	
<2	1 (2.0)
2-5	5 (9.8)
5-10	17 (33.3)
10-15	16 (31.4)
15-18	4 (7.8)
18-29	4 (7.8)
≥30	4 (7.8)
Age at diagnosis, Mean (SD)	3.5 (2.7)
Years since diagnosis, y (n = 47)^b^	
Mean (SD)	9.3 (8.4)
Median (min, max)	6.8 (0.7, 46.9)
Severe patients	
Mean (SD)	6.7 (5.4)
Median (min, max)	5.2 (0.7, 27.5)
Attenuated patients	
Mean (SD)	14.3 (11.0)
Median (min, max)	11.9 (1.7, 46.9)
Family history of MPS II	
Yes	17 (32.7)
If yes, prenatal diagnosis	1 (5.9)
No	31 (59.6)
Do not know	4 (7.7)
MPS II Classification, n (%)^c^	
Severe (with cognitive impairment)	36 (69.2)
Attenuated (without cognitive impairment)	16 (30.8)
Living Situation, n (%)	
Living with parents	48 (92.3)
Living alone	3 (5.8)
Living in an institution during the week	1 (1.9)

^a^Data missing for 1 patient.

^b^Data missing for 5 severe phenotype patients.

^c^Estimates of the relative prevalence of each MPS II phenotype at any point in time are affected by the higher mortality rates for severe versus attenuated patients, and may vary substantially.

Of the 52 patients, 36 (69.2%) were considered to have the severe phenotype with cerebral involvement and 16 (30.8%) had the attenuated form, based on their physician’s diagnosis. The MPS-associated medical conditions and surgical interventions reported among all patients and within each phenotype are shown in Table 2. The most common medical conditions for all patients were: hearing loss (41 out of 51 respondents, 78.8%); disabling joint stiffness (40/52, 76.9%); and hernia (37/52, 71.2%). About 81% (42/52) of all patients, and similar proportions of severe and attenuated patients (30/36 and 12/16, respectively) had experienced at least 1 surgical intervention, the most common being ear ventilation tubes (20/42 [47.6%] total; 13/30 [43.3%] severe, 7/12 [58.3%] attenuated). Four (25.0%) attenuated patients had experienced mild mental disability without cognitive decline.

**Table 2:** **Serious MPS II disease manifestations and concomitant medical issues for the patients in the study by phenotype**

**Disease manifestation** ^**a**^ **, n (%)**	**Severe (n = 36)**	**Attenuated (n = 16)**	**Total (N = 52)**
Hearing loss	28 (75.7)	13 (86.7)	41 (78.8)
Disabling joint stiffness	25 (69.4)	15 (93.8)	40 (76.9)
Hernias	24 (66.7)	13 (81.3)	37 (71.2)
Adenoidectomy	22 (61.1)	13 (81.3)	38 (67.3)
Mental retardation	24 (66.7)	4 (25.0)	28 (53.8)
Spine deformities	17 (45.9)	9 (60.0)	26 (50.0)
Tonsillectomy	16 (44.4)	9 (56.3)	25 (48.1)
Recurrent otitis	17 (45.9)	7 (46.7)	24 (46.2)
Loss of vision	11 (30.6)	8 (50.0)	19 (36.5)
Sleep apnoea	13 (36.1)	5 (31.3)	18 (34.6)
Skin involvement	11 (30.6)	4 (25.0)	15 (28.8)
Other serious clinical events^b^	10 (27.0)	3 (20.0)	13 (25.0)
Breathing difficulties with hospitalisation	9 (25.0)	4 (25.0)	13 (25.0)
Stay in intensive care unit^c^	5 (13.9)	3 (18.8)	8 (15.4)
Aspiration requiring hospitalisation	2 (5.6)	1 (6.3)	3 (5.8)
Spinal cord compression	1 (2.7)	1 (6.3)	2 (3.8)
Heart failure with hospitalisation	1 (2.7)	1 (6.7)	2 (3.8)

^a^Counts are not mutually exclusive, patients can have more than 1 manifestation.

^b^Behavioural problems (6), asthma (1), epilepsy (1), psoriasis (1), accelerated growth (1), Prader-Willi syndrome (1), mitral valve dysplasia (1).

^c^1 post-operatively, and 1 for breathing problems.

#### Medical service needs

Hospital, medical, and paramedical consultations received during the past 12 months of the study are shown in Table 3. Of 51 respondents, 26 (51.0%) had been admitted to hospital because of their MPS II disease, with a mean length of stay of 10.4 days (range 1–120 days). Thirteen (25.5%) patients had visited the emergency room a mean number of 2 times due to MPS II during the previous 12 months, most commonly for respiratory symptoms (6/13, 46.2%). The medical specialists visited in the past year by the greatest number of patients were dentists (22/51, 43.1%); ear, nose, and throat specialists (25/51, 49.0%); and ophthalmologists (15/51, 29.4%).

**Table 3:** **Hospital, medical and paramedical visits/consultations related to MPS II during the past 12 months (N = 51)**

**Hospital visits**	**n (%)**	**Mean (SD) visits/y**
Emergency Room Visits	13 (25.5)	2.0 (1.3)
Respiratory problems	6 (46.2)	
Abdominal pain/diarrhoea	2 (15.4)	
Ear infection	2 (15.4)	
Fever	2 (15.4)	
Chest pain	1 (7.7)	
Septic shock	1 (7.7)	
Admitted to hospital	26 (51.0)	1.3 (0.8)
Duration of stay (days)		10.4 (25.3)
**Medical specialty**	**n (%)**	**Mean (SD) Visits/y**
Dentist	22 (43.1)	4.3 (6.4)
Ear, nose, and throat	25 (49.0)	11.2 (22.0)
Ophthalmologist	15 (29.4)	1.5 (1.6)
Orthopaedic surgeon^a^	14 (29.8)	2.7 (2.5)
Other specialist	16 (31.4)	
Dermatologist	5 (9.8)	
Pneumonologist	4 (7.8)	
Cardiologist	4 (5.9)	
Allergologist	1 (2.0)	
Paediatrician	1 (2.0)	
**Paramedical specialty**	**n (%)**	**Mean (SD) Visits/mo**
Physical therapist	43 (84.3)	7.4 (4.9)
Speech therapist	20 (40.0)	5.3 (2.3)
Psychomotor therapist	21 (45.7)	4.0 (1.8)
Psychologist	14 (32.6)	2.8 (1.4)

^a^n = 47.

### Clinical Management

#### MPS II diagnosis

Among the 44 patients for whom diagnosis data were available, the mean age at diagnosis was 3.5 ± 2.7 years, ranging from < 1 year of age for 4 (9.1%) patients to 10 years and over for 3 (6.8%) patients. As noted above, although a family history of MPS II was ultimately found to be present in 17/52 (32.7%) of patients, prenatal diagnosis had been performed for only 1 patient. As of the time of the study, the mean time since diagnosis was 9.3 ± 8.4 years. There was a substantial disparity in the mean time since diagnosis among severe patients (6.7 years) versus attenuated patients (14.3 years): twice as long for attenuated compared with severe patients, as would be expected based on the contrasting life expectancy in the 2 groups.

The first MPS II symptoms occurred before the age of 1 year in most MPS II patients and at a mean age of 2.0 ± 2.3 years. The first symptoms leading to suspicion of MPS II were observed by parents or child for 25 (49.0%) of the 51 respondents. In 7 (13.7%) instances, the treating paediatrician or family doctor first suspected the condition, and for 6 (11.8%) patients, identification occurred incidentally during a medical consultation or hospitalisation. Five patients were initially suspected to have MPS II because a family history of MPS II had been documented, and 6 were identified by the school physician or speech therapist. Among 47 patients responding, the time from observation/documentation of the initial symptoms to formal diagnosis of MPS II (or diagnosis delay) was less than 3 years for 10 (21.3%) patients, 3 to 7 years for 14 (29.8%), and from 7 to 20 years for 19 (40.4%) patients. While the majority 29/47 (61.7%) were diagnosed within 9 years from the first appearance of symptoms, 3/47 patients were diagnosed after 20 to 45 years, and 1 patient waited more than 45 years for diagnosis. The largest proportion of patients (22/51, 43.1%) had to see 2 to 4 physicians before the diagnosis was made, while one-third (17/51) saw only 1 physician, and 9 patients consulted with 5 to 8 physicians before receiving their final diagnosis. With regard to the manner and sensitivity of the delivery of the final diagnosis, 20 (40.0%) out of the 50 respondents felt the diagnosis was delivered in a brusque fashion, 17 reported the delivery was tactful, and 13 said it was given with no special consideration. Psychological support was suggested following the delivery of diagnosis to only 14/49 (28.6%) respondents, while the majority (40/50, 72.0%) received no such recommendation or referral. Only half of the 50 respondents reported that they had received clear information about the condition, and a minority (19/50) described the information given as “sufficient”.

#### Place of idursulfase treatment

Idursulfase treatment took place in several locations. For the 51 patients with data, 32 (62.7%) were treated at a nearby hospital or infusion center, 13 (25.5%) at regional specialised centers for rare diseases (“Centres de Compétence”), and 4 (7.8%) patients were treated at home (1 patient given home nursing care and 3 given hospital care at home) under the *Hospitalisation à Domicile* (HAD) program. Two patients discontinued treatment because it was considered to be ineffective (the patient’s condition worsened).

#### Treatment regimen

The mean total time of idursulfase treatment, incorporating both periods (from baseline of initial treatment until the present) was 3.8 ± 1.3 years for this population; the median total time was 4.3 (range 0.5–7.3) years. The mean idursulfase dose was 0.6 ± 0.2 mg/kg, and the frequency of infusion was once per week in 50 (96.2%) patients. The mean time of infusion per treatment was 2.3 ± 0.6 hours.

#### Home versus hospital treatment

When asked if they would prefer treatment at home under HAD, 30 (63.8%) of 47 respondents responded “yes”; the others responded “no” or “undecided”. Twenty-six of the 37 respondents who marked “yes” or “undecided” and responded to the follow up question thought that home administration would benefit them because it is more comfortable for the patient to stay in their own environment, and 23 said it eliminated the need to go to the hospital every week. Eleven respondents of the 15 who wrote “no” or “undecided” reported that they found hospital care reassuring, 6 said they wanted to avoid having their home become more like a hospital, and 4 felt that medical monitoring of infusion was important. Other objections expressed to home treatment included difficulty adapting the home for the service, the belief that socialization to hospital conditions was important, or that the hospital was better able to help control a child’s agitation.

All of the 4 patients, 2 adult patients and 2 families with their children, who were at the time having their treatment administered by the HAD program, reported satisfaction with the organization and conduct of their care. When asked for the reason for their satisfaction, 3 respondents said they preferred to stay in their own environment, and 2 said that it saved them from travelling, 2 each stated that it made for better family arrangements and eliminated the need to go to hospital for treatment, and 1 reported that it saved time.

### Impact of MPS II on quality of life

MPS II had a distinct impact on various aspects of QoL and living arrangements for families. With regard to work, among 44 respondents, 20 (45.5%) had to reorganize their working hours to attend to patient care, and 10 (22.7%) reported that 1 of the 2 parents had to stop working, 4 (9.1%) had to change work, 1 had to take repeated time off from work, and 1 had to take parent leave. Mothers were far more frequently affected in their working life than fathers (37 versus 5, respectively). Of the 7 patients who were of working age, 3 had never worked because of their long-term illness, 2 were working part time, 1 worked irregularly/casually, and 1 was at college and lived in a special needs medical/educational facility.

MPS II also affected place of residence for 4 of 51 families, with treatment considerations taking precedence over other preferences, and necessitating a return to France from abroad in 1 case. Of the 51 respondents, 8 (15.7%) had paid home help, at an average duration of 8.5 hours/week, while 18 (35.3%) families received regular help from volunteers or relatives. The remainder of respondents said they did not need outside help, stating they would look after their family and their home, in spite of the situation “like everyone else”.

#### QoL scale results

Only 14 respondents (9 parents, 5 patients) completed the KIDSCREEN-27 questionnaire, including 7 each from the severe and attenuated groups. Parents were the respondents for all 7 severe patients, while among the attenuated respondents, 5 were patients and 2 were parents. The KIDSCREEN-27 scores for the MPS II population were consistently lower (worse) compared with the reference control population for all 5 dimensions of the scale (physical well-being, psychological well-being, autonomy and parents, peers and social support, and school environment), regardless of age group and of whether parents or patients were the respondents. Patient/parent scores generally ranged from 30 to 60, while reference population scores ranged from 60 to 90, with median scores usually in the 75 to 85 range, across all dimensions. No individual patient/parent scores reached the reference population median score for any dimension. The few instances where individual respondent scores were within the lowest quartile of the reference population scores occurred with 1 patient respondent aged between 8–11 years for the dimensions of autonomy and parents, and school environment; 1 patient aged between 12–18 years for peers and social support, and school environment, and another patient in this age group for school environment; and 2 parent scores, 1 for peers and social support, and the other for school environment.

Five completed EQ-5D questionnaires were received and analysed, all from adults answering for attenuated patients; although 12 parents of severe patients responded to the questionnaire, none completed all the questions. Results for the EQ-5D VAS, which is for adults only and covers 5 dimensions (mobility, autonomy, current activities, pain/discomfort, and anxiety/depression), were lower than those for the reference population, as observed with the KIDSCREEN-27. All total respondent EQ-5D scores ranged from about 50 to 70, while reference population scores ranged from approximately 75 to 90.

### Support Services Utilization

#### Social services and financial support

Of 51 respondents, 37 (72.5%) reported they used a social services assistant to help them with the administrative procedures required to request financial assistance, or a home care aide, or to find a specialized facility for patient care. Twelve (23.5%) respondents used the services related to their Reference Center for Rare Disease, and 12 (23.5%) used services from schools and specialized centers.

Three quarters of 51 respondents (n = 39 [76.5%]) received an education financial allowance for disabled children (*Allocation d’Education de l’Enfant Handicapé* [AEEH]), 15 (29.4%) received a disability living allowance (*Prestation de Compensation du Handicapé* [PCH]), 6 adult patients received an adult disability allowance, and 2 (3.9%) received a compensatory allowance for a third person (*Allocation Compensatrice pour Tierce Personne* [ACTP]). Among the 39 families receiving the AEEH benefits, 16 received no other aid, while 13 also received PCH. In addition, 10 (19.6%) families received various other types of financial aid. Only 3 families received no financial aid.

#### Psychological counseling

Of the 51 respondents, only 7 (13.7%) indicated they had received professional psychological counseling. Thirty-seven (72.5%) were members of a patients’ association, all of whom were members of *Vaincre les Maladies Lysosomales*; 5 were also members of another association. The benefits of belonging to an association, according to these respondents, included obtaining information, interacting with other families/patients, social contact, making useful contacts, obtaining help, listening, and support.

#### Travel and other costs

Travel was one of the major costs for families. The distance traveled to centers for follow up evaluation and medical treatment ranged widely, from < 20 kilometers (km) for 10 (20.0%) patients to more than 700 km for 6 (12.0%) patients. The majority of patients (66.7%) used their car or a taxi for these trips. The 19 (37.3%) families who traveled more than 400 km, mainly represented patients who lived in the Southwest of France or Brittany and used the center at Lyon for annual evaluation. This helps explain why 7 (13.7%) families typically traveled by airplane and 6 (11.8%) by train to the infusion centers. The distance traveled for weekly infusions was 30 km or less for the majority of families (65.6%), with only 1 patient needing to travel more than 100 km; 90.7% of families used their car or a taxi for these trips.

Travel costs were reimbursed for 89.8% of patients, although 13/52 (25.0%) families chose not to file for reimbursements for short journeys made to medical and paramedical appointments. Five families declined this benefit entirely because they found the administrative process too complicated and restrictive, and 3 had not even thought of going through the process. Seventeen families were reimbursed for beds, 9 families had paid for travel to paramedical consultations and to the infusion center, and 5 indicated that accommodations during tests were an additional expense. Two families required transport for a second adult to accompany them during tests, and 2 families required travel to medical and educational special needs facilities. Nine families indicated that they had to pay for adaptations to their home or car.

One respondent indicated that hospital parking was an additional cost. Other items listed as additional costs paid by families included hearing aids, wheelchairs, and non-reimbursable drugs.

#### Choice of centers for follow up care

Twenty-seven (54.0%) patients had a choice of center for follow up care. Notwithstanding the substantial travel costs, only of 13 (48.1%) of these 27 based their choice primarily on the proximity of the center to their home. Other common criteria for the choice of follow up center included a referral by a third party (29.4%), because it was the center where the patient had been diagnosed (23.5%), and familiarity with the medical staff at the follow up center (25.9%), or with the center in general (18.5%), among others. Overall, 32 (62.7%) of 51 respondents used different centers for follow up and infusion.

### Idursulfase CGI-I and PGI-I results

#### CGI-I

Among all 52 patients, severe and attenuated combined, 44 (84.6%) reported that they were either “much improved”, or “minimally improved”, 4 (7.7%) patients showed “no change”, and 2 (3.8%) were judged “much worse” during Period 1. For Period 2 (end of the first year of treatment to the time of study evaluation), among all patients, 24 (46.2%) were “very much improved”, “much improved”, or “minimally improved”. The 1 patient who was rated “very much improved” was aged 8.5 years and had an attenuated form of MPS II. In addition, 18 (34.6%) patients were rated “minimally worse”, “much worse”, or “very much worse”, and 8 (15.4%) patients were rated unchanged.

Analysis of CGI-I scores by phenotype is shown in Table 4. For Period 1, among the 33 severe patients, 93.9% were rated either improved (minimally-, much-, or very much improved [n = 10; 30.3%]) or unchanged (n = 21; 63.6%), while only 2 (6.1%) were minimally-, much-, or very much worse. Of the 16 attenuated patients, all (100%) were either improved (n = 7; 43.8%) or unchanged (n = 9; 56.3%). For Period 2, the majority of severe patients (51.5%) were unchanged, while only 3 (9.1%) were improved and 13 (39.4%) were worse. Response during Period 2 was more favorable among the attenuated patients, with 7 (43.8%) patients rated as improved, 7 (43.8%) rated as unchanged, and only 2 (6.1%) rated as worse. Differences by age in CGI-I scores were observed during Period 1, with mean (SD) age at start of treatment being younger in severe patients who improved compared with those who remained stable or worsened (4.0 [1.6] versus 6.8 [5.3] and 6.9 [3.7] years, respectively) and in attenuated patients who were improved or remained stable (11.1 [6.6] versus 19.5 [13.0] years, respectively). During Period 2, mean (SD) age at start of treatment among severe patients was also younger for those improved compared with those stable or worsened (3.1 [2.8] versus 5.9 [5.9] and 6.2 [2.4] years, respectively). Among attenuated patients, mean (SD) age was younger in improved patients than in stable patients, but was also younger in worsened than in stable patients (11.0 [7.8] versus 21.6 [13.4] versus 12.6 [5.5] years, respectively). Multivariate analysis showed no statistically significant relationship between age at start of treatment or at diagnosis and CGI-I scores for either Periods 1 or 2 and for either phenotype, probably due to small sample sizes.

**Table 4:** **CGI-I results for patients with severe and attenuated phenotypes and for the total population for 2 time periods**

	**Severe (n = 33)**			**Attenuated (n = 16)**			**Total population (N = 52)** ^**e**^	
**Period 1: From baseline (ERT onset) versus 1 y later**	**n (%)**	**Mean age at start of ERT (y)**	**Mean age at diagnosis (y)**	**n(%)**	**Mean age at start of ERT (y)**	**Mean age at diagnosis (y)**		**N (%)**
Significant improvement^a^	10 (30.3)	4.0	2.6	7 (43.8)	11.1	4.4	Improvement^f^	44 (84.6)
No change/little improvement or worsening^b^	21 (63.6)	6.8	2.4	9 (56.2)	19.5	6.5	No change	4 (7.7)
Significant worsening^c^	2 (6.1)	6.9	2.7	0 (0)	–	–	Worsening^g^	2 (3.8)
*P* value^d^		0.1181	0.9448		0.1248	0.2235		
**Period 2: From end of first year ERT versus present**	**n (%)**	**Mean age at start of ERT (y)**	**Mean age at diagnosis (y)**	**n (%)**	**Mean age at start of ERT (y)**	**Mean age at diagnosis (y)**		
Significant improvement^a^	3 (9.1)	3.1	2.1	7 (43.8)	11.0	4.9	Improvement^f^	24 (46.2)
No change/little improvement or worsening^b^	17 (51.5)	5.9	2.7	7 (43.8)	21.6	6.8	No change	8 (15.4)
Significant worsening^c^	13 (39.4)	6.2	1.7	2 (12.5)	12.6	3.7	Worsening^g^	18 (34.6)
*P* value^d^		0.2619	0.0638		0.1710	0.6233		

^a^“Very much improved” and “much improved”.

^b^“Minimally improved”, “no change”, and “minimally worse”.

^c^“Much worse” and “very much worse”.

^d^Wilcoxon test.

^e^Two patients in the total population were not evaluable each during Period 1 and Period 2.

^f^“Very much improved”, “much improved”, and “minimally improved”.

^g^“Minimally worse”, “much worse”, “very much worse”.

#### PGI-I

**Figure 1:**
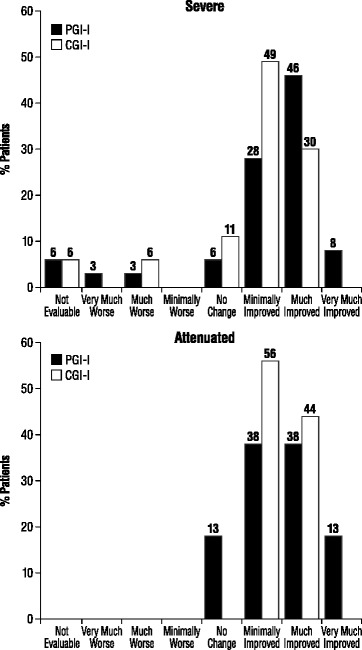
Comparison of the CGI-I and PGI-I responses for patients with severe and attenuated forms of MPS II for Period 1 (from before ERT to after 1 y of treatment). Severe PGI-I: n = 33, CGI-I: n = 33. Attenuated PGI-I: n = 16, CGI-I: n = 16.

**Figure 2:**
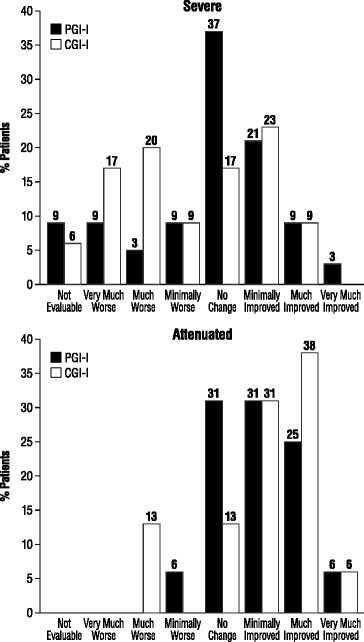
Comparison of the CGI-I and PGI-I responses for patients with severe and attenuated forms of MPS II for Period 2 (1 y after start of ERT to present^a^). Severe PGI-I: n = 33, CGI-I: n = 33. Attenuated PGI-I: n = 16, CGI-I: n = 16. ^a^Final measurements taken January 31, 2012. Period from end of the first year of treatment to the present varied among patients but was >2 years.

For Period 1, severe patients/caregivers gave highly positive PGI-I responses, with 54.0% stating they were either “very much improved” (8.0%) or “much improved” (46.0%), while 28.0% said they were “minimally improved”; 6.0% were unchanged and 3.0% each were much worse or very much worse (Figure 1). Among the attenuated patients/caregivers, the PGI-I responses for Period 1 were also strongly positive, with 50.0% stating they were either very much improved (12.5%) or much improved (37.5%), while 37.5% said they were minimally improved and 12.5% were unchanged; no attenuated patients/caregivers said the condition had worsened. For Period 2, the PGI-I ratings were somewhat less positive, with the greatest percentage of severe patients/caregivers (37.0%) rating the patient’s condition as unchanged (Figure 2). However, one-third (33.0%) of the severe cohort still rated themselves/the patient as very much improved (3.0%), much improved (9.0%), or minimally improved (21.0%); 12.0% of severe patients were rated worsened compared with 6% during Period 1. Among attenuated patients, most patients/caregivers (62.0%) rated themselves/the patients as either minimally improved (31.0%) or unchanged (31.0%); 31.0% were rated very much improved (6.0%) or much improved (25.0%), and 6.0% were worsened, compared with 0% during Period 1.

## Discussion and conclusions

This study gathered broad data on the impact and burden of MPS II, the organization and efficiency of clinical management for this disease, and the benefits of ERT for MPS II, in France. The study included 52 patients being treated with idursulfase, representing 83.9% of all such eligible patients that had been identified at French Reference Centers for Metabolic and Lysosomal Diseases. The patients represented a wide range of ages (from 1 year to 51 years), and the majority (69.2%) had a severe phenotype, which is consistent with other epidemiologic estimates of about two-thirds of patients with the severe phenotype in sample MPS II populations at any given point in time [2]; however, the higher mortality rates of severe relative to attenuated patients may render such prevalence estimates considerably variable. It should also be noted that as respondents were asked to recollect current and historical data, the likelihood of recall bias cannot be eliminated.

In this trial, we chose to include only treated MPS II patients. At first glance, this may appear as a major weakness of the study; however, at the time of the study, as therapy was available commercially in France, the number of untreated patients was extremely limited and most of them were at a very advanced stage of the disease, some of them being also under palliative care. Given this setting, it appeared that including untreated patients as a comparison group would have introduced a greater degree of bias into the study than excluding them. It seems likely that if all patients with MPS II had been included in the study then the burden of disease may have been recorded as being greater than is shown here.

The well-known, multisystemic disease manifestations and problems associated with MPS II were represented in this study population [2,19]. Large majorities of both severe and attenuated patients had experienced hearing loss, disabling joint stiffness, hernia, and adenoidectomy at the time of the study. The frequency of various medical conditions among the attenuated patients, in some cases exceeding that in the severe group, may be explained by the greater time since diagnosis and the older ages of the attenuated patients. Hence, despite the clear differences between the phenotypes, these data also illustrate how attenuated patients can ultimately be as greatly affected as severe patients by many of the somatic disease manifestations of MPS II over their longer life spans. Although cognitive impairment is more commonly associated with the severe phenotype [2,3], 4 (25.0%) patients with the attenuated phenotype had experienced mild mental retardation without regression, and about 80% of both severe and attenuated patients had experienced at least 1 surgical intervention.

Difficulties in clinical management of MPS II were also apparent from these data. A family history of MPS II was ultimately found to be present in 17 (32.7%) patients, although only 1 prenatal diagnosis had been performed. However, the majority of parents and patients did not know of the existing MPS II cases in their family at the time of diagnosis and these family histories were only discovered retrospectively. The majority of patients did not know of the existing MPS II cases in their family. Identification of (and commensurate attention to) first symptoms was more commonly made by parents (49.0% of cases) than by the treating paediatrician or family doctor (13.7% of cases), which may indicate a need for better education in recognition of the signs and symptoms of MPS II among paediatrician and family doctors. Indeed, about 40% of patients had to wait from 7 to 20 years for a diagnosis following the initial observation and documentation of symptoms, and 9 (17.6%) patients/families had to see from 5 to 8 physicians. Although data are lacking on rates of recognition of MPS II among clinicians, it has been noted that expertise in diagnosis and management of MPS II varies between countries in Europe [20]. In addition, 40% of respondents felt the diagnosis, when delivered, was given in a *façon brutale* or brutal way, and psychological support was offered at the time of diagnosis to only 28.0% of respondents. Finally, only half of respondents felt that they had received clear information about the disease upon diagnosis from the diagnosing physician. Overall, the survey data on detection and diagnosis of MPS II suggest more should be done to disseminate knowledge of this rare disease among clinicians and thus facilitate rapid and compassionate diagnosis, and timely treatment.

Twenty-two out of 33 patients reported they received idursulfase “immediately” after diagnosis. We consider this a positive feature of the French healthcare system, which allows initiation of ERT in the weeks following MPS II diagnosis. Infusions were the most commonly administered at hospital (62.7%), while only 4 (7.8%) patients were treated at home under the HAD program, although 64.0% of patients/families said they would like to receive home infusions. Home infusion cannot be performed in France unless a hospital facility provides the drug and infusion. The majority of families who decided against home infusion, however, said they found hospital care reassuring. A potential bias is that patients on home therapy may see a physician less frequently, so potential complications may be detected less promptly, in comparison with those patients seen frequently in hospital settings. This might lead to an improvement in perceived QoL over and above that of idursulfase treatment for those in a hospital setting. On the other hand, patients who are able to receive ERT at home are likely to be more stabilized, so this may help counter this bias. The authors believe; however, that disease evaluation and treatment in France is essentially the same, whether patients receive ERT in hospital or at home, and that therefore improved QoL is unlikely to be linked to the site of administration of ERT.

The impact of MPS II on patient/family QoL came out in responses to the patient/parent questionnaire and the results of the KIDSCREEN-27 and EQ-5D scales. Close to half of parents (45.5%) had to reorganize their working hours to accommodate patients care, and about one-fifth (22.7%) had to stop working. Choice of place to live was affected for 4 families, and many families had either obtained paid household help (15.7%) or were receiving regular help from volunteers or relatives (35.3%). The many, diverse demands of MPS II for healthcare, as listed in Table 3 (i.e., hospital, medical specialist, and paramedical specialist visits and services), suggest some of the likely reasons for these lifestyle adjustments. However, approximately half of the families were willing to manage their needs without seeking household help. On both the KIDSCREEN-27 and the EQ-5D scales, respondent scores were well below median reference population scores across all dimensions and age groups of respondents. The mean total EQ-5D scores ranged from 5 to 40 VAS units lower than those of the reference population; the minimally important difference for the EQ-5D VAS has been estimated to range from 7–12 [21]. In a recent study, Raluy-Callado et al. showed, using a number of validated patient and caregiver questionnaires, that MPS II patients with the attenuated phenotype generally score consistently lower than paediatric subjects with type 1 diabetes, juvenile idiopathic arthritis, asthma, or attention deficit disorder [4]. Physical function and the ability to perform day-to-day activities were the most affected areas in a group of patients when compared with normal paediatric populations. Health-related QoL issues were also impacted by the disease, particularly the psychological aspects such as self-esteem and family cohesion, where the subjects scored even lower than those for physical function. This emphasis on the impact of the disease on caregivers and families, in particular, agrees with the findings from this study. Likewise, the hearing loss experienced by 80.0% of the patients in this study is in agreement with previous work [21,22]. Although the KIDSCREEN-27 and EQ-5D results suggest that the QoL of patients with MPS II is lower than the reference population, the number of respondents was low, and definitive conclusions cannot be reached from these QoL measures in this study. The authors’ experience is that KIDSCREEN-27, in particular, is not well adapted to assessing patients with severe MPS II.

Use of social and financial support benefits was extensive. Almost all families received some form of financial support, most commonly (76.5%) the education allowance for disabled children (AEEH) available in France; only 3 families received no financial support. Many also received social services support (72.5%), mostly to help with financial aid (73.0%) and social service paperwork (86.5%). This level of support demonstrates how much these patients benefit from healthcare support in France, particularly when compared with many other countries. Travel costs and accommodations during tests were also a substantial financial burden for families, for which they often did not seek reimbursement either out of choice or because they found the application process too cumbersome. These data on the experience of families in coping with the burden of MPS II, and the social structures available to help them, suggest that more can be done to increase the number of patients receiving home infusion, which was a preferred option for most patients/families and could also decrease travel costs. Better access to educational and other social facilities for children is also needed for MPS II patients.

With regard to response to treatment, the CGI-I questionnaires showed that after the first year of ERT (Period 1), 93.9% of severe patients were either improved (n = 10; 30.3%) or stabilized (n = 21; 63.6%), while only 2 (6.1%) worsened, and all 16 attenuated patients showed either improvement (n = 7; 43.8%) or stabilization (n = 9; 56.3%) (Table 4). Among patients with either phenotype, the best improvement was observed in patients with the youngest mean (SD) age at the start of ERT (Table 4). Further improvement during Period 2 (from the end of the first year of ERT to the present) was seen by clinicians in 3 (9.0%) severe patients, while 51.5% were stable and 39.4% worsened; as in Period 1, the improved severe patients had a younger mean age than stable or worsened patients. These results are consistent with the natural history and progression of MPS II in severe patients. Among attenuated patients, 87.6% were either improved (n = 7; 43.8%) or stable (n = 7; 43.8%) after Period 2, while only 2 were worse, for reasons which were not specified but could have been related to factors beyond treatment effect (e.g., neurosurgery). Overall, given the progressive nature of MPS II, particularly in severe patients, it is clinically significant and could be regarded as beneficial that 9.1% of severe patients and 43.8% of attenuated patients manifested CGI-I improvement in status during Period 2, after a mean of 3.8 ± 1.3 years of ERT. The multivariate analysis showing neither age at diagnosis nor age at the start of idursulfase treatment was associated with CGI-I ratings, among both severe and attenuated patients, and during both treatment periods, was surprising since MPS II is generally less responsive to treatment after disease progression, but this may be due to insufficiently large sample sizes. Nonetheless, the nonsignificant associations of younger age with better response to ERT are consistent with our previous experience that this treatment is most effective for somatic problems at earlier rather than late stages of progression.

The disparities between the PGI-I and CGI-I ratings during both Periods 1 and 2, shown in Figures 1 and 2, are also notable. In the severe group, the percent of patients/parents rating the patient “very much improved” or “much improved” during Period 1 was almost double that of clinicians giving these ratings (55.0% combined ratings versus 30.3% combined ratings, respectively). Perhaps a placebo effect influenced this result. Conversely, in the attenuated group, more clinicians than patients/caregivers rated the patients “much improved” or “minimally improved” (44.0% and 56.0% versus 38.0% for each rating, respectively) for Period 1; it could be that the clinicians had a more positive view than the patients because they tend to compare them with the more rapid disease progression of the severe phenotype. In addition, the patients may experience treatment-related burdens that give them a more negative impression of their condition. However, 13.0% of PGI-I ratings for Period 1 were “very much improved” compared with 0% of CGI-I ratings for the same period, which could also indicate a placebo effect for some patients. For Period 2, the CGI-I and PGI-I ratings were somewhat more consistent with each other; however, more than twice the percentages of patients/caregivers rated the patient as unchanged in both the severe and attenuated groups versus the clinician ratings, and more clinicians than patients/caregivers saw worsening in the patients during this period. Overall, it must be noted for both the CGI-I and PGI-I that the ratings for Period 1 were given retrospectively since most patients had been receiving treatment for several years (mean 3.8 years), introducing a bias of perspective from the present time to the first-year period. In addition, the treatment intervals differ for each patient, which could further influence the ratings. Therefore, given the variability of patient factors and treatment conditions from which these retrospective, observational data were drawn, the study results must be interpreted with caution.

Drawn from a “real-world” clinic sample, this study adds to the increasing knowledge about the characteristics and burden of MPS II, its clinical management in France, and the effects of idursulfase treatment from both the clinician and patient perspectives. It was the intention of this study, and it is the aspiration of the authors, that these data help to clarify the full burden of MPS II on patients and their families, and also to identify areas for improvement in the clinical management of this disease and in providing support for families in coping with it.

## Abbreviations

ACTP: Allocation compensatrice pour tierce personne
CGI-I: Clinical global impression-improvement
EQ-5D: EuroQoL-5D
ERT: Enzyme replacement therapy
HAD: Hospitalisation à Domicile
L’AEEH: L’Allocation d’Education de l’Enfant Handicapé
MPS: Mucopolysaccharidosis
PCH: Prestation de compensation du handicap
PGI-I: Patient global impression-improvement
QoL: Quality of life
VAS: Visual analogue scale
